# A Case of Traumatic Tetanus Treated by Physostigma

**Published:** 1878-09

**Authors:** 


A Case of Traumatic Tetanus Treated by Physostigma.—Charles G., æt. 7, thin in flesh, of a nervous temperament. September 17, 1877, while playing, he climbed on a fence, and jumping off, his left foot struck a broken bottle that was concealed in the grass. The glass cut through the fascia, muscles and external plantar artery, making a wound about one inch long. This was dressed by the family, hemorrhage being arrested by binding cobweb on the foot.

On September 21st I first saw the boy. He was sitting propped up in bed, complaining of severe pain in the foot, which was much swollen. The toes were almost black from the effects of the tight bandage, which had not been removed since it was dressed. The entire leg was red and swollen. He complained of pain running up the inside of the leg. I at once removed the bandage, and as the hemorrhage was very free, I gave chloroform, and ligated the posterior tibial artery, behind the internal malleolus. As this only partly controlled the hemorrhage, I ligated the dorsalis pedis, which arrested it entirely. I then examined the wound in the foot, and found the skin, fascia and muscles in a gangrenous state, and sloughing for a space of two inches in circumference. The foot was ordered to be poulticed with slippery elm, and an opiate was administered; I also gave him two grains citrate of quinine and iron every four hours.

Sept. 22. Rested well through the entire night; foot swollen very badly; the swelling in the leg subsiding and not painful; continued the poultice, changing every four hours, and washed the sloughing wound with carbolic acid solution one part to twenty. The boy remained much the same until the 26th, when the skin and the muscles of the foot that were gangrenous sloughed off, leaving a granulating surface two by three inches in extent. I continued the citrate of quinia and iron, but he did not require the opiate. He ate well until the last twenty-four hours, since when he has eaten nothing; ordered beef essence and dressed his foot with vaseline, and gave him a dose of castor oil.

27th, 6 A. M. He was very restless all night, and continually turned over from side to side; slept but little; opens his mouth with difficulty and swallows poorly; is unable to move without great pain; pulse 90 and weak; bowels freely opened by oil taken last night; perspires freely; expression of face anxious; complains of spasmodic pain in the back, epigastrium, groins and calves. Touching his hands or any part of the body produces a short clonic spasm. He was ordered to take ten grains of bromide of potassium, and five grains of chloral hydrate every three hours; beef essence, one egg and one ounce of brandy every four hours.

1 P. M. Resting well; has no pain; touching hand or body does not cause spasms, but the muscles are more rigid. Continued the same treatment.

8 P. M. He has had within the last hour three severe spasms, lasting half a minute, each causing opisthotonos. The perspiration very profuse; the eyes fixed; the pupils widely dilated; respirations very distressing; muscles of neck and jaw rigid, producing in the face the characteristic ghastly grin; pulse 100 and weak; is quite sensible, and his sufferings consequently severe. I now ordered tinct. cannabis indicæ, twelve drops (to be increased two drops at each dose) to be given with the bromide and chloral until he became quiet.

28th, 6 A. M. He is much worse in every respect. The spasms are more severe and last longer; has had twenty-three since last note, some lasting fully one minute; the wound on the foot is dry, there not being a drop of pus; pulse 105 and very weak; respiration 32 and difficult; perspiration in drops over the entire body; pupils widely dilated and do not respond to light.

The bromide and chloral combined with the tinct. cannabis indicæ, having done no apparent good, were discontinued, but the beef essence, egg and brandy were given as before, although he had great difficulty in swallowing. I now prescribed physostigma in one-eighth grain doses every two hours. He took the first dose at 6:30 A. M. At

11 A. M., when I saw him, the pupils were of normal size, the spasms not so hard, and the pulse 99. Continued the physostigma. At 3 P. M., has had but one spasm since 12 o'clock, that being a very severe one; swallows better, and does not perspire so freely. Continued the physostigma, beef essence, egg and brandy. 9:30 P. M. The pupils are about the size of pin heads; has had two very light spasms, lasting about ten seconds each; to take the physostigma every three hours.

29th, 7 A. M. Slept for three hours last night, then woke up in a light spasm at 1 A. M., since which he has had several short naps, but no spasms; can swallow much better; pupils size of small pin heads; muscles rigid; respirations 24 and not so difficult. Continued beef essence, egg and brandy; to take physostigma every three and one-half hours. 1 P. M. Slept for two hours, and woke up in light spasm; pupils not so small as last noted; he is perspiring more freely, and is more nervous. Ordered physostigma to be given every three hours. 9 P. M. Has had three naps since last note, awoke each time more nervous, but had no more spasms; muscles very rigid, pupils contracted; respirations 24 and somewhat difficult; expresses himself as feeling much better.

30th, 7 A. M. Had no spasm last night, and from this date on he had no spasms. I continued the physostigma at various intervals, as he appeared nervous, until Oct. 14. He made a slow and tedious convalescence, the rigidity of the muscles lasting until the last of October, so much that it prevented him from walking. The treatment from this date consisted of tonics and stimulants, with nutritious food. Complete recovery ensued.

